# Increased Adipogenic and Decreased Chondrogenic Differentiation of Adipose Derived Stem Cells on Nanowire Surfaces

**DOI:** 10.3390/ma7042605

**Published:** 2014-03-28

**Authors:** Nathan A. Trujillo, Ketul C. Popat

**Affiliations:** Department of Mechanical Engineering, Colorado State University, Fort Collins, CO 80523, USA; E-Mail: nathan.trujillo@rams.colostate.edu

**Keywords:** nanowire surfaces, polycaprolactone, adipose derived stem cells, chondrogenic differentiation, adipogenic differentiation

## Abstract

Despite many advances in tissue engineering, there are still significant challenges associated with restructuring, repairing, or replacing damaged tissue in the body. Currently, a major obstacle has been trying to develop a scaffold for cartilage tissue engineering that provides the correct mechanical properties to endure the loads associated with articular joints as well as promote cell-scaffold interactions to aid in extracellular matrix deposition. In addition, adipogenic tissue engineering is widely growing due to an increased need for more innovative reconstructive therapies following adipose tissue traumas and cosmetic surgeries. Recently, lipoaspirate tissue has been identified as a viable alternative source for mesenchymal stem cells because it contains a supportive stroma that can easily be isolated. Adipose derived stem cells (ADSCs) can differentiate into a variety of mesodermal lineages including the adipogenic and chondrogenic phenotypes. Biodegradable polymeric scaffolds have been shown to be a promising alternative and stem cells have been widely used to evaluate the compatibility, viability, and bioactivity of these materials. Polycaprolactone is a bioresorbable polymer, which has been widely used for biomedical and tissue engineering applications. The fundamental concept behind successful synthetic tissue-engineered scaffolds is to promote progenitor cell migration, adhesion, proliferation, and induce differentiation, extracellular matrix synthesis, and finally integration with host tissue. In this study, we investigated the adhesion, proliferation, and chondrogenic and adipogenic differentiation of ADSCs on nanowire surfaces. A solvent-free gravimetric template technique was used to fabricate polycaprolactone nanowires surfaces. The results indicated that during the growth period *i.e*., initial 7 days of culture, the nanowire surfaces (NW) supported adhesion and proliferation of the cells that had elongated morphologies. However, cell on surfaces without nanowires had non-elongated morphologies. Further, immunofluorescence imaging of marker proteins showed that the nanowires surfaces did not appear to support chondrogenic differentiation whereas supported adipogenic differentiation of ADSCs.

## 1. Introduction

Stem cells possess three unique characteristics: self-renewal capacity, long-term viability, and multilineage potential [1,2]. There are two types of stem cells that exist in the body: somatic stem cells and embryonic stem cells. The ability of somatic stem cells and embryonic stem cells to differentiate into various cell phenotypes has been studied extensively. Embryonic stem cells are pluripotent cells, which have the ability to differentiate into all derivatives of the three germ layers. Although they possess great multilineage potential, research associated with embryonic stem cells is accompanied with many ethical and political issues [1,2]. Due to that fact, mulitpotent somatic stem cells from the bone marrow stroma have been proposed as the best alternative source for research in the past few decades. These adult stem cells from the bone marrow stroma are classified into two groups: hematopoietic stem cells (HSCs) and mesenchymal stem cells (MSCs). Originally, MSCs were identified as a source of osteoprogenitor cells, however it has been shown that they can also differentiate into adipocytes, chondrocytes, osteoblasts, and myoblasts [3–8]. Their ability to differentiate into different phenotypes makes them promising candidates for mesodermal defect repair and disease management. However, clinical use of MSCs in regards to tissue repair has presented problems including pain due to the invasiveness of the procedure of isolation, morbidity, and low cell number upon harvest. This has led many researchers to investigate alternate sources for MSCs.

Recently, studies have shown that adipose tissue is believed to contain an abundant source of multipotent progenitor cells [9]. Subcutaneous adipose tissue is a highly abundant and readily accessible tissue source in the body [10]. Thousands of liposuction surgeries are performed in the U.S. each year and these procedures yield anywhere from 100 mL to >3 L of lipoaspirate tissue [11]. Adipose tissue, like bone marrow, is derived from the mesenchyme and contains a supportive stroma that can be easily isolated. Although it remains to be determined whether adipose derived stem cells (ADSCs) meet the same potential as that of MSCs, it is known that ADSCs are multipotent, available in large numbers from the adipose tissue, and proliferate rapidly in culture, making them an attractive cell source for tissue engineering. *In vitro*, adipose derived stem cells (ADSCs), like MSCs, can differentiate into multiple cell lineages including osteogenic, chondrogenic, myogenic, adipogenic and even neuronal pathways [9]. In addition, ADSCs have also demonstrated a substantial *in vitro* bone formation capacity, similar to that of MSCs from bone marrow, with much ease of culture [2,12,13].

In tissue engineering, MSCs or ADSCs are often implanted or seeded onto artificial biomaterials known as scaffolds, which provide the necessary structure for growth, maintenance, and differentiation of the cells in the early stages of tissue repair. In regards to polymer scaffold materials, polycaprolactone has emerged as a promising implant material due to the fact that it is biodegradable polyester with good mechanical strength and a low degradation rate [14]. It also has low melting point between 60 and 65 °C, and is derived by chemical synthesis from crude oil and can be prepared by ring opening polymerization of caprolactone using a catalyst [14]. It has good water, oil, solvent and chlorine resistance but can be degraded by hydrolysis of its ester linkages in physiological conditions and its degradation products are easily bioresorbed or removed naturally in metabolic pathways such as the citric acid cycle [14]. Its efficacy has resulted in US-FDA approval for a number of medical devices. Recently, it has received a much attention for use as an implantable biomaterial for tissue engineering and drug delivery applications [15–18]. Studies have shown that polycaprolactone is biocompatible *in vitro and in vivo* [19]. For example, it is regarded as a soft and hard tissue compatible bioresorbable material [20] and has been considered as a potential substrate for wide applications such as drug delivery systems [21], tissue-engineered skin [22] axonal regeneration [23] and scaffolds for supporting fibroblasts and osteoblasts growth [24].

It is important to mimic the *in vivo* environment of cells when designing scaffolds for tissue engineering. The motivation to use nanostructured surfaces as scaffolds for tissue engineering is driven by previous studies that have shown that nanoscale materials affect cell behavior such as morphology, functionality and cell-cell interactions [25–27]. In natural tissues, cells are surrounded by an extracellular matrix, which consists of features ranging from nanometers to micrometers. Furthermore, studies have shown that nanoscale surfaces contributed to improving cell behavior such as fibroblast adhesion [28], neuronal differentiation [29], and osteoblast phenotypic activity [30,31]. Therefore, nanotopography may result in improved cellular adhesion and thus, enhanced matrix deposition on the surface for other cell types such as chondrocytes and adipocytes. It could also be possible that these nanostructured surfaces, which are not able to allow cellular in-growth due to their size, will instead, provide a biomimetic template for matrix deposition.

Cartilage tissue possesses a unique nanostructure rarely duplicated in synthetic materials. Specifically, chondrocytes are naturally accustomed to interacting with a well-organized nanostructured collagen matrix [32]. The unique microarchitecture of extracellular cartilage matrix facilitates the load transfer and provides resistance to tensile, compressive, and shear stresses. Unlike other tissues, in hyaline cartilage, roughly 85% consists of extracellular matrix materials while only 15% are taken by chondrocytes [33]. The extracellular matrix overtakes the biomechanical function of the cartilage and the small number of cells only responsible for its preservation and regeneration [33]. Similar to osteoblast interaction with surrounding surfaces *in vitro*, chondrocyte functionality is also heavily regulated by surface micro and nano topography *in vitro*. The most common nanostructured materials developed for chondrogenic tissue engineering applications are nanofiber scaffolds. Recent studies have used synthetic polymers such as polycaprolactone and poly (lactic-co-glycolic acid) to develop nanofibrous scaffolds of electropun nanofibers to differentiate MSCs to chondrocytes and maintain their mature functionality [34]. In addition, polycaprolactone nanofiber scaffolds have been used in conjunction with submersion in a dioxane/water mixture and phase separated to create a nanoporous structure [35]. These polycaprolactone nanoporous scaffolds demonstrated higher *in vitro* expression of chondrogenic markers and had higher histological scored in comparison with commercially available collagen scaffolds typically used for cartilage repair [35].

In addition to osteogenic and chondrogenic differentiation, adipocyte differentiation is a complex process regulated by many transcriptional cascades and hormonal stimuli. [36–40]. One important aspect of adipocyte differentiation is the role of extracellular matrix proteins and cytoskeleton proteins [41]. Because these proteins have a unique structure, it is very likely that extracellular matrix micro and nanoscale topography would affect adipogenesis in *in vitro* culture conditions on a biomimetic scaffold. Previous studies have demonstrated that adipogenic differentiation can be upregulated to a certain extent by changing the geometry to the scaffold to features such as nanogrooves [42]. Cells seeded on nanogroove surfaces penetrated into the grooves with the actin cytoskeleton being more aligned along the grooves, suggesting that cell-to-surface interactions as characterized by contact guidance are closely related to the adipogenesis [42]. In addition, studies have shown that tailored electroactive nanorods and hydrophobic nanopillars have been successful in not only differentiating MSCs into adipocytes, but actually increasing the rate of differentiation in comparison with flat surfaces [43,44].

In this study, we use a solvent-free gravimetric template method to physically modify the surface of polycaprolactone with controlled arrays of high aspect ratio substrate-bound perpendicular nanowires. In this study, these nanowire surfaces were used as templates for growth and maintenance of ADSCs, and their potential to support differentiated states of these cells. Specifically, this study focuses on characterizing the ability of ADSCs to differentiate into chondrogenic and adipogenic phenotypes on nanowire surfaces.

## 2. Results and Discussion

Despite many advances in tissue engineering, there are still significant challenges associated with restructuring, repairing, or replacing damaged tissue in the body. Currently, a major obstacle has been trying to develop scaffolds for cartilage tissue engineering that provides the correct mechanical properties to endure the loads associated with articular joints as well as promote cell-scaffold interactions to aid in extracellular matrix deposition. In addition, adipogenic tissue engineering is widely growing due to an increased need for more innovative reconstruction therapies following adipose tissue traumas and cosmetic surgeries. Recently, lipoaspirate tissue has been identified as a viable alternative source for MSCs because it contains a supportive stroma that can easily be isolated. ADSCs can differentiate into a variety of mesodermal lineages including the adipogenic and chondrogenic phenotypes. This study is aimed at determining the effects of substrate bound nanowires surfaces on the viability and differentiation potential of ADSCs into chondrogenic and adipogenic lineages.

**Figure 1.**
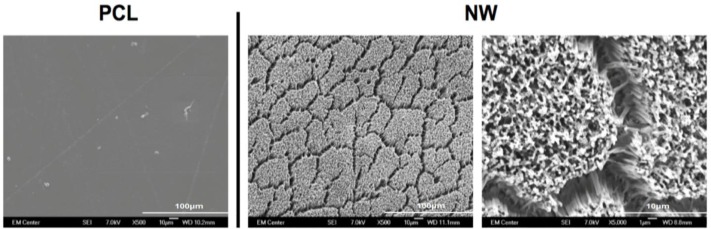
Representative SEM images of polycaprolactone discs (PCL) and polycaprolactone nanowire surfaces (NW). The high magnification SEM images of NW show the nanoarchitecture as well as presence of randomized microchannels.

**Figure 2.**
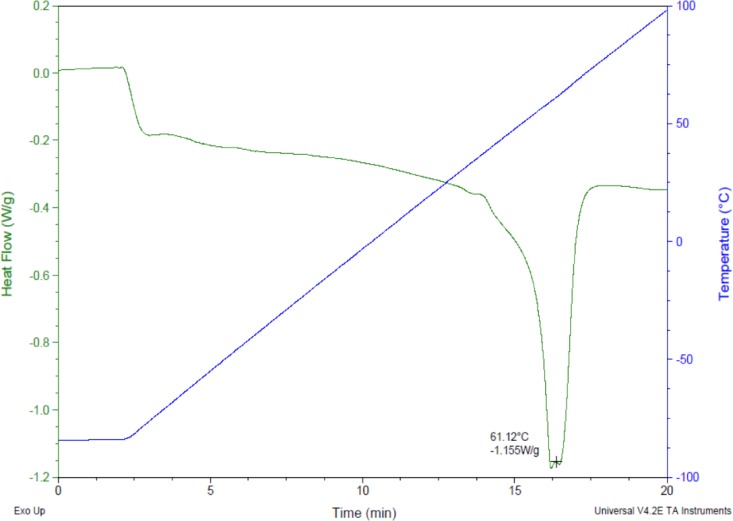
Differential scanning calorimetry (DSC) of polycaprolactone displaying the melting temperature of 61.12 °C.

Prior research with polycaprolactone NW surfaces have shown the ability of stem cells to differentiate into osteogenic lineages [45]. In this study, a similar sintering and solvent-free nanotemplating method was used to fabricate both PCL and NW. SEM images confirmed the presence of substrate bound perpendicular nanowires as well as the absence of residual alumina membrane (Figure 1). SEM images also indicated randomized microchannels between the groups of nanowires due to static surface charge and surface tension following expansion of the nanowires after membrane dissolution. DSC determined that the melting temperature of 50,000 Da polycaprolactone powder was 61.12 °C (Figure 2). To ensure there would be adequate extrusion of the polymer through the aluminum oxide membrane during nanowire fabrication, the polymer was melted slightly above the melting temperature at 70 °C for 20 min when the formation of a clear melt interface was present between the bulk material and the membrane.

**Figure 3.**
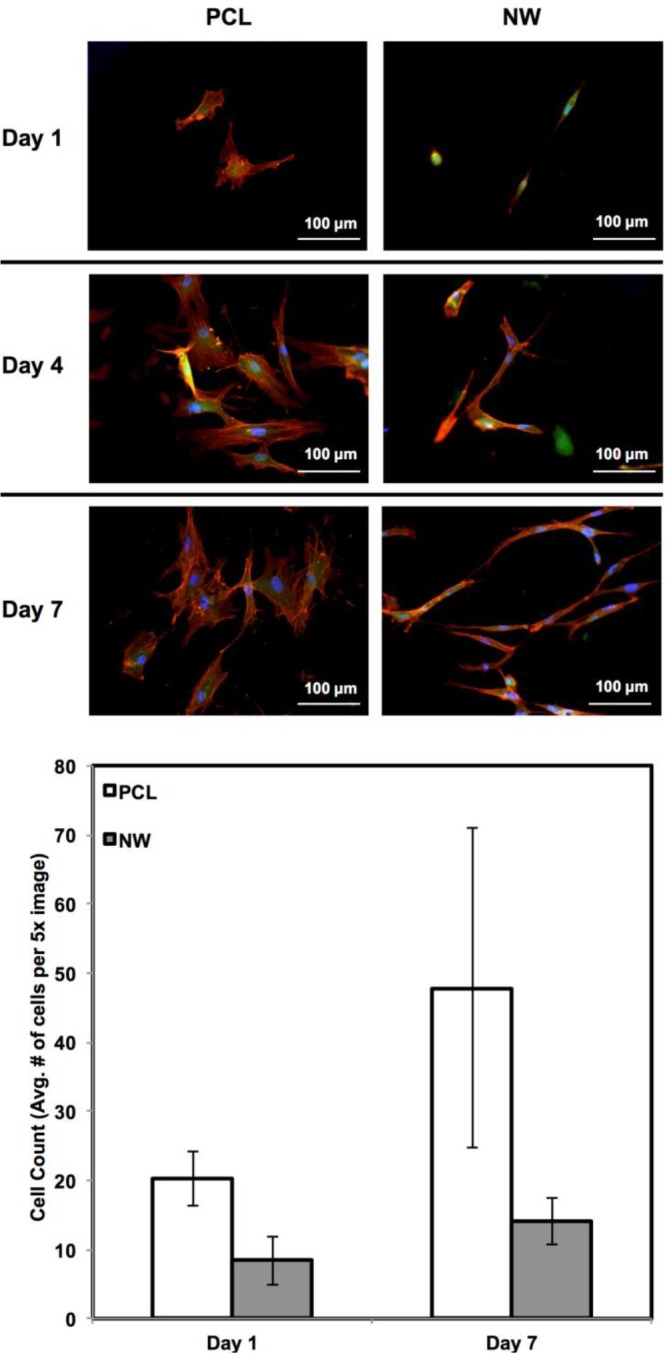
Representative fluorescence microscopy images of ADSCs on PCL and NW after 1, 4, and 7 days of culture; cell count after 1 and 7 days of culture. Cell nuclei were counted using DAPI fluorescence and ImageJ software [45].

ADSC adhesion and proliferation were investigated for up to 7 days of initial culture in growth media by staining the cells with CellTracker™ Green CMFDA (5-Chloromethylfluorescein Diacetate), Rhodamine-phalloidin and DAPI (Figure 3). CMFDA fluorescent probes are FITC labeled which causes them to fluoresce green as they freely pass through the membranes of viable cells. Once these probes reach the cytoplasm, the probes are converted to cell-impermeant reaction products which are present throughout the cell. Once this reaction takes place, the cells can be fixed using a formalin solution. Rhodamine-phalloidin is a high affinity F-actin probe coupled with a red-orange fluorescent dye called tetramethylrhodamine (TRITC). DAPI is a DNA stain which binds to the adenine-thymine rich regions and causes the nuclei of the cells to fluoresce blue. After 1 day of culture, both surfaces supported similar cell adhesion with different cell morphologies. On PCL, the cells appear to have “round” morphologies due to the actin filaments organizing themselves in all directions. On NW, the cells have elongated morphologies because the actin filaments of each cell appear to align in a specific direction with the nanowires. After 4 and 7 days of culture, both surfaces support cell proliferation with the slightly more cells on PCL as compared to NW (Figure 3). However, although NW have lesser number of cells, their extended morphologies appear to form networks with neighboring cells using cellular extensions.

**Figure 4.**
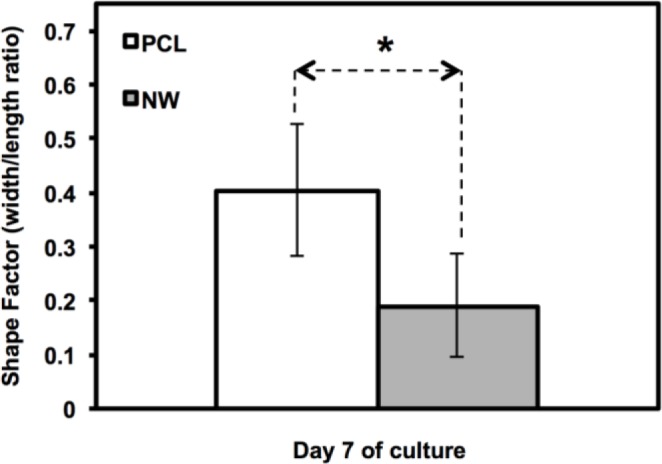
Shape factor approximations of Adipose derived stem cells (ADSCs) after 7 days of culture on PCL and NW. Asterisks (*) above the pairs of data indicate statistical significance using p-value less than α = 0.05.

The cell morphology was partially quantified by calculating the cell shape factor using 10× rhodamine-phalloidin stained images after 7 days of culture (Figure 4). The shape factor is an important quantitative parameter that indicates the whether the cell is spherical or elongated. A shape factor→1 indicated that the cell has spherical morphology, whereas shape factor→0 indicated that the cell has elongated morphology. The shape of the cell is important since it not only affects cell migration and communication, but also the cell differentiation. The results indicate that the cell on PCL have shape factor higher than that on NW. This indicated that the cells have spherical morphology on PCL compared to elongated morphology on NW.

**Figure 5.**
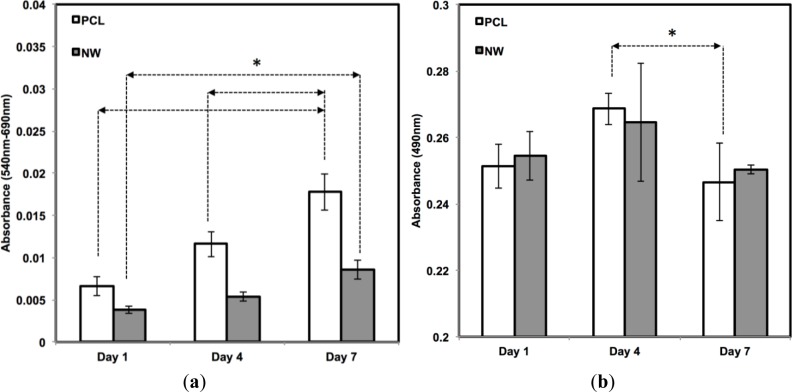
(**a**) ADSC viability after 1, 4, and 7 days of culture using MTT absorbance. Data indicates increased MTT activity on all surfaces with significant increases on PCL; (**b**) Cytotoxicity of the surfaces after 1, 4, and 7 days of culture using LDH absorbance. Asterisks (*) above the pairs of data indicate statistical significance using p-value less than α = 0.05.

The cell viability on all the surfaces was investigated using MTT assay after 1, 4, and 7 days of culture in growth media conditions (Figure 5a). MTT activity is measure of cell viability by quantifying the absorbance of mitochondrial activity. During the first 4 days of culture, mitochondrial activity is most likely directed at increasing the proliferation of cells across the surfaces. After 1 day of culture, the results indicate approximately an equal amount of MTT activity on both surfaces. Although both surfaces continue to have steady and significant increases from day 1 to day 7, there appears to be more MTT activity on the PCL in comparison with NW which confirms the results from the fluorescence microscopy images and cell counts. The number of cells that initially attach to PCL after one day of culture is double in comparison with NW and this correlates with the MTT results.

In addition to observing cell viability, it is also essential to determine whether the surfaces have any cytotoxic effects that may be inhibiting cell adhesion or proliferation. Material cytotoxicity was determined through an LDH assay (Figure 5b). LDH is an enzyme located inside the cytoplasm of cells that is released upon cell death. This assay measures the amount of formazan following a two-step reduction, where LDH catalyzes nicotinamide adenine dinucleotide (NAD+) to nicotinamide adenine dinucleotide hydride (NADH) and H+ by oxidation and a subsequent catalyst reaction by diaphorase converting tetrazolium salt to a colored formazan. Measurements using spectrophotometery determine the concentration of LDH released into the culture medium, or in other words, determine the level of cell death due to possible cytotoxic effects from the substrate. From day 1 to day 4, there is an increase in LDH absorbance, due to initial cell death and detachment from the surfaces in the first 4 days of the growth period. However, this absorbance level decreases from day 4 to day 7, which means that both surfaces, PCL and NW, are not cytotoxic to the cells over time.

**Figure 6.**
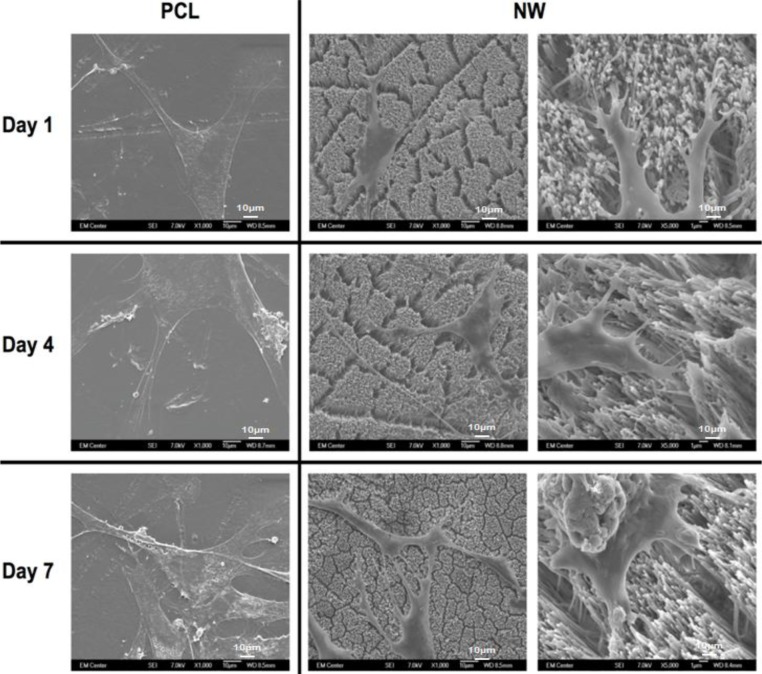
Representative SEM images of ADSCs on PCL and NW after 1, 4, and 7 days of culture. The high magnification images on NW show cell extensions and adhesion sites between the ADSCs and the individual nanowires.

To visualize the morphological changes in the cells on different surfaces, SEM images were taken after 1, 4 and 7 of culture (Figure 6). In addition to morphology, SEM also allows for visualization of the interaction of the cell processes with the surface nanoarchitecture as well as amongst cells. The SEM results support the results from fluorescence microscopy indicating higher number of cells on PCL than NW, as well as elongated cell morphologies on NW. The cells on PCL seem to spread in all directions with limited interaction with other cells. In contrast, the cells on NW have longer cellular extensions that seem to be involved in cell to cell communication. High magnification SEM images of cells on NW show that the cellular extensions are interacting with the nanowire architecture after 1 day of culture. These extensions seem to be longer after 7 days of culture showing the interaction of the cells with the nanowire architecture as well as form a complex cellular network.

**Figure 7.**
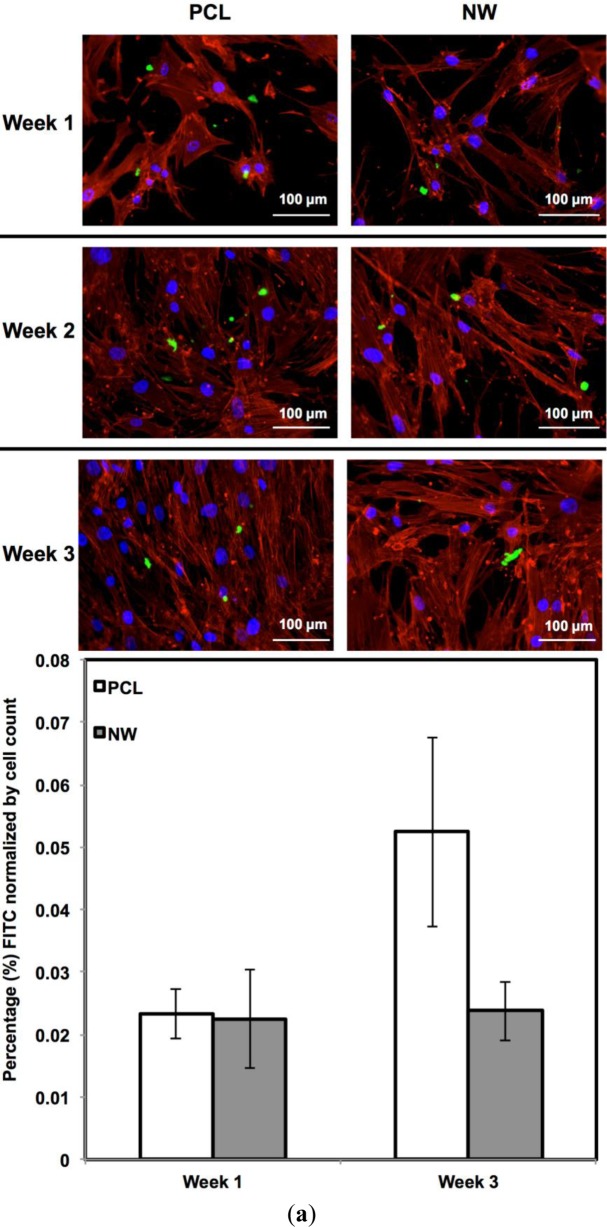

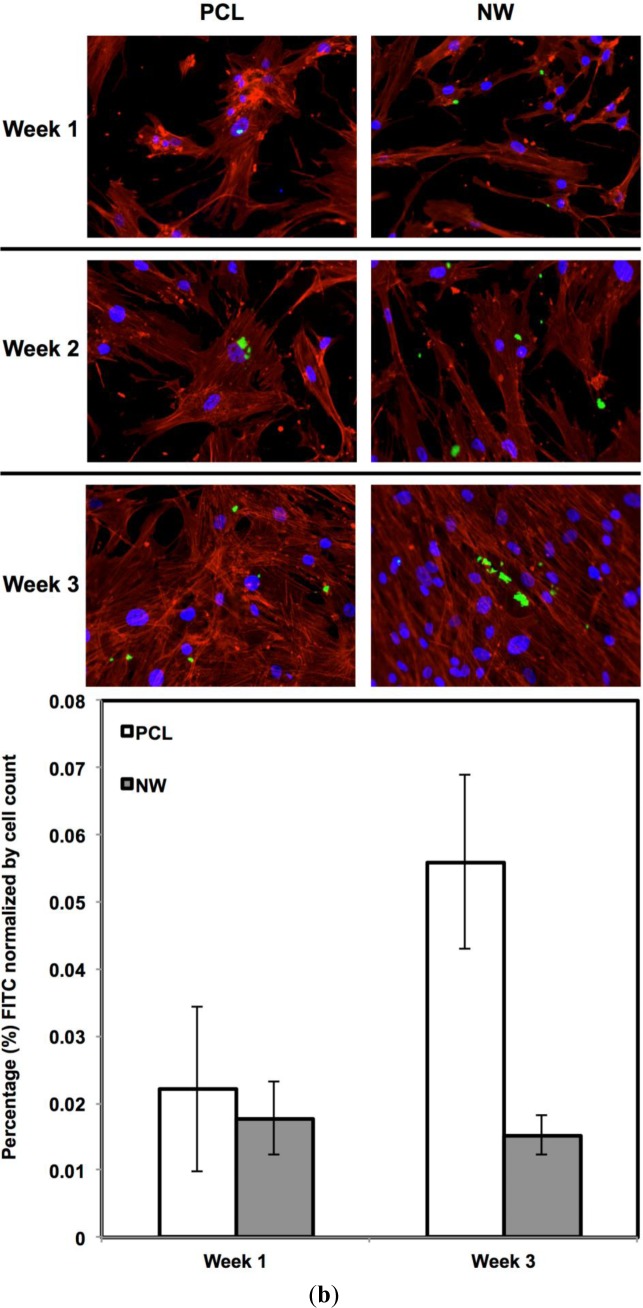
(**a**) Representative immunofluorescence images of ADSCs on PCL and NW for sox9 (green), actin (red) and nuclei (blue) after 1, 2, and 3 weeks of culture in chondrogenic conditions; (**b**) Representative immunofluorescence images of ADSCs on PCL and NW for col2 (green), actin (red) and nuclei (blue) after 1, 2, and 3 weeks of culture in chondrogenic conditions. Percentage of FITC-labeled sox9 (**a**) and col2 (**b**) were normalized by total number of cells within a particular image.

For surfaces cultured under chondrogenic differentiation conditions, ADSCs on the different surfaces were immuno-labeled for sox9 and col2 (collagen type II) (Figure 7a,b). Sox9 is a protein transcribed by the sox9 gene within the nucleus and is an early marker for differentiation as well as a marker for mature chondrocytes. Although sox9 is present during most of the chondrogenic differentiation process, it is not expressed by cells undergoing hypertrophic chondrogenesis. Col2 is the basis for all articular cartilage and is a marker for maturing chondrocytes. Intracellular sox9 and intra/extracellular col2 was detected by FITC-labeled immunofluorescence after 1, 2 and 3 weeks ADSCs culture under chondrogenic conditions.

The percentage of FITC detected during immunofluorescence was normalized by the number of cells on the particular image (Figure 7a,b). For sox9 expression, the protein was present on both surfaces during all three weeks of differentiation. However, ADSCs on PCL saw an increase from week 1 to week 3 whereas ADSCs on NW saw the same amount of expression. Even though the percentage of FITC does not increase from week 1 to week 3 on NW, the amount of sox9 protein increases because the number of cells on the surfaces increased. A similar trend is observed for col2 expression on both surfaces. However, for both sox 9 and collagen 2, this increase protein expression on NW is significantly smaller in comparison to the cells on PCL. It appears that the nanotopography of NW does not have a significant effect on chondrogenic differentiation of ADSCs.

**Figure 8.**
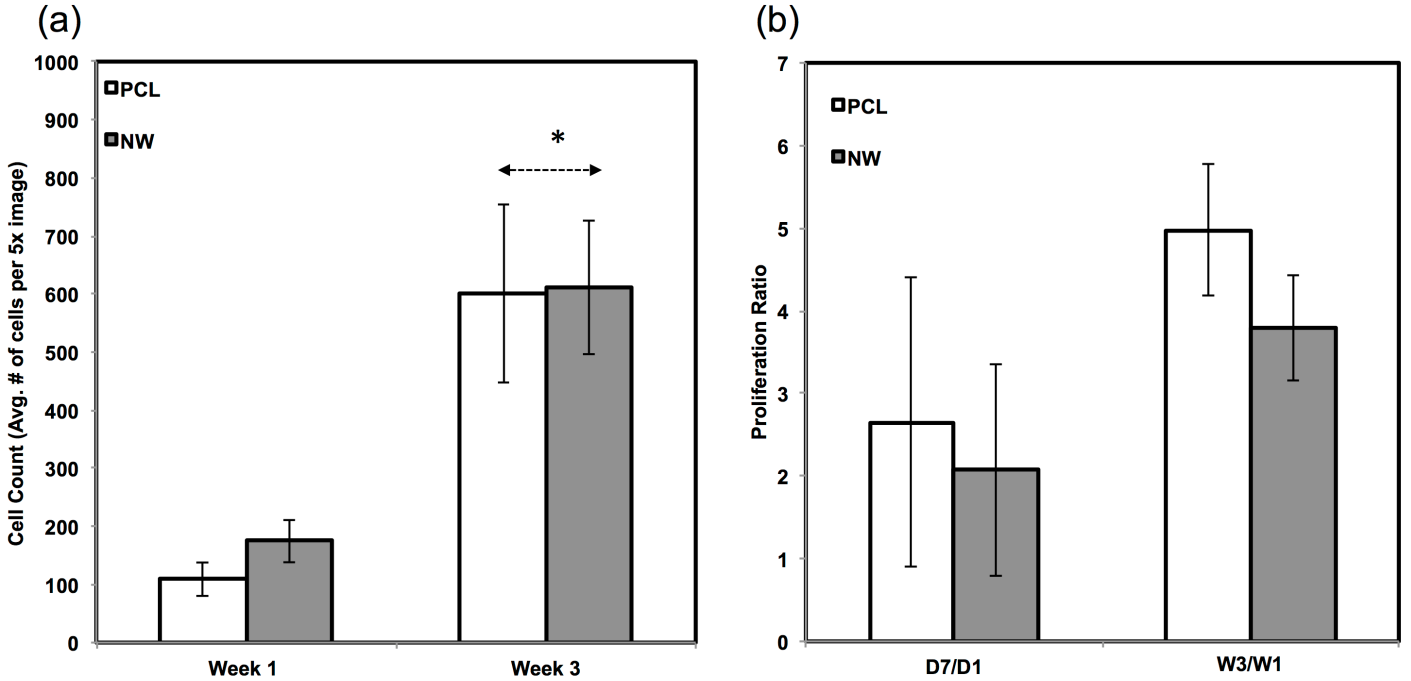
(**a**) Average cell counts on PCL and NW after 1 and 3 weeks of culture in chondrogenic conditions. Cell nuclei were counted using DAPI fluorescence and ImageJ software [45]; (**b**) Comparisons of proliferation ratios between the growth period (D7/D1) and the differentiation period (W3/W1). Asterisks (*) above data pairs indicate statistical significance using p-value less than α = 0.05.

Since the chondrogenic differentiation media was supplemented with a high concentration of TGF-β1, there was increased proliferation on both PCL and NWfrom week 1 to week 3 (Figure 8a). The proliferation ratio for PCL during initial growth period (D7/D1) is lower to that during the differentiation period (W3/W1) (Figure 8b). Further, the proliferation ratio of NW during growth period (D7/D1) is also lower than that during the differentiation period (W3/W1) (Figure 8b). This increase in proliferation is due the presence of TGF-β1 in the differentiation media.

**Figure 9.**
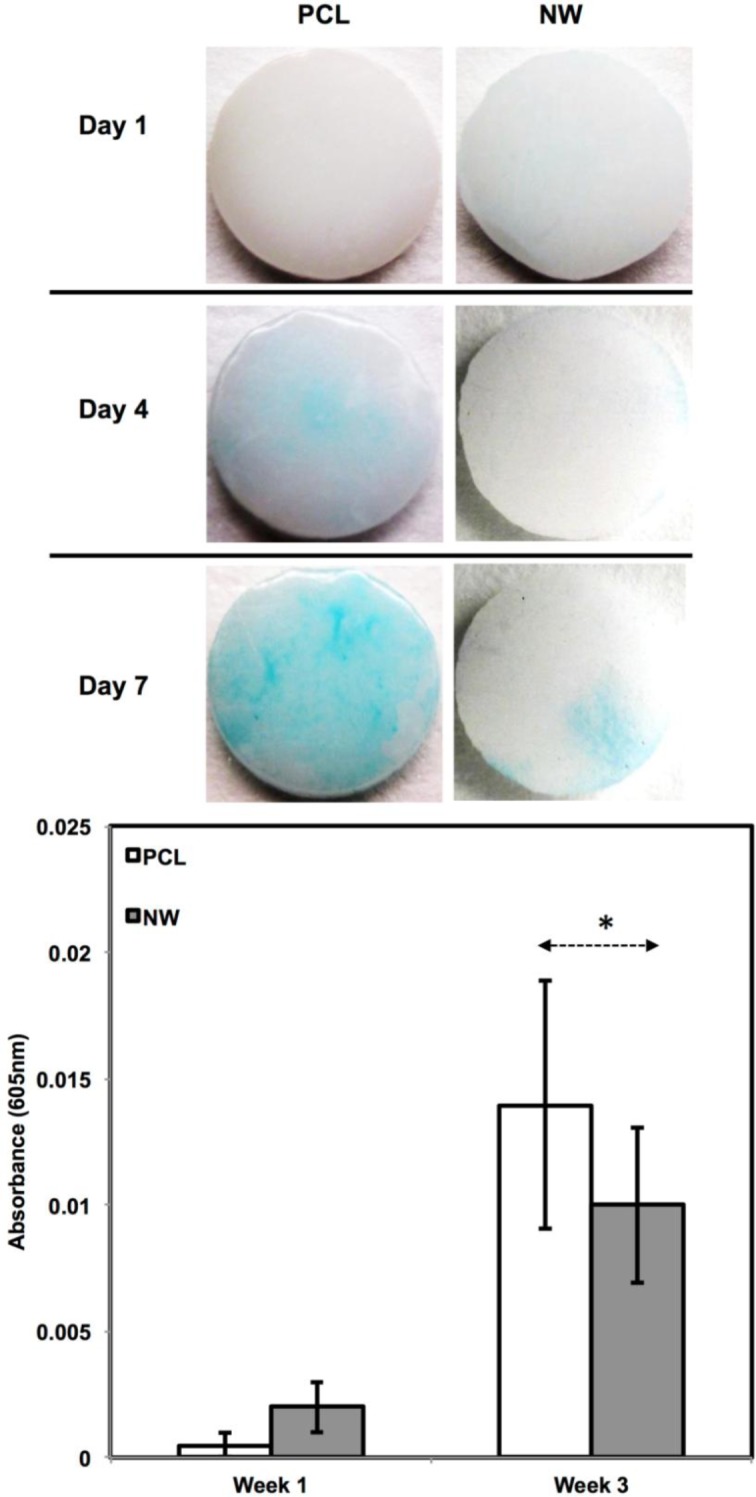
Alcian blue (GAG) staining of PCL and NW after 1, 2 and 3 weeks of culture in chondrogenic conditions as well as quantification of alcian blue absorbance after 1 and 3 weeks of culture. Asterisk (*) above Week 3 indicates statistical significance from Week1 using p-value less than α = 0.05.

In addition to observing protein expression via immunofluorescence, ADSCs were stained using alcian blue for early differentiation and extracellular matrix production (Figure 9). Alcian blue is a polyvalent dye that is used to stain for acidic polysaccharides such as glycosaminoglycans (GAGs) and sulfated glycosaminoglycans (sGAGs). These polysaccharides are widely present in all connective tissues and especially in both articular and hyaline cartilage tissue. After 1 week in chondrogenic differentiation conditions, both surfaces had little to no staining of alcian blue, however it is evident after 3 weeks in chondrogenic differentiation conditions, both surfaces showed alcain blue staining indications that GAGs were expressed on the surface. alcian blue staining followed the same trend that was observed during sox 9 and col2 immunofluoresnce. Although both surfaces showed that GAGs were present, the PCL had much more alcian blue absorption than NW and therefore, more GAG production. Although the photos give a comparable qualitative measure of the stain, the alcian blue was solubilized in 1% SDS to give a quantitative measure of the dye using spectrophotometry. As expected, both surfaces saw an increase in alcian blue absorption from week 1 to week 3, but the adsorption on NW was less than PCL.

To further understand the behavior of the ADSCs as they are subjected to chondrogenic differentiation, it is important to look at the role of cytoskeletal organization and cytoskeletal stress. Although spreading and aligning of the actin filaments of cells on NW and within the NW microgrooves is advantageous for osteogenesis to occur [46,47], typical culture methods for inducing chondrogenic differentiation are done in suspension which involves little stress on the cells [48,49]. Unlike the method used in this study, the cells pellets do not endure high cytoskeletal tensile stresses like they do on NW surfaces. Since the cells are more isotropic on the PCL, there is more chondrogenic differentiation than the cells that have aligned filaments on the NW.

**Figure 10.**
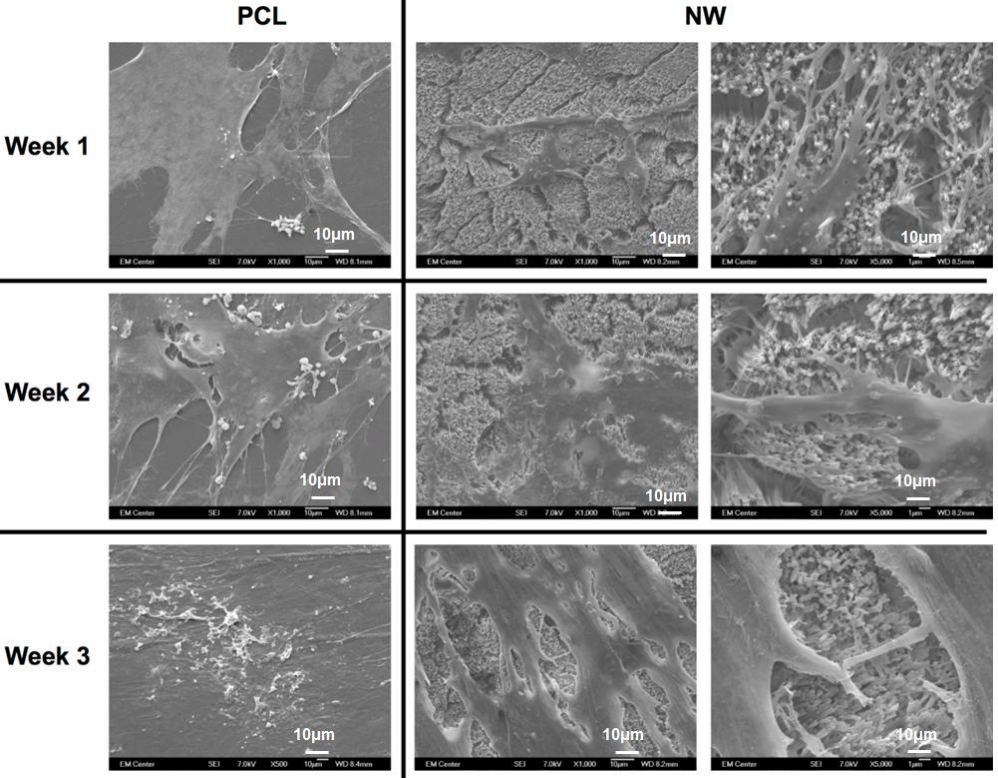
Representative SEM images of ADSCs on PCL and NW after 1, 2, and 3 weeks of culture in chondrogenic conditions.

The cell morphology after 1 and 3 weeks of chondrogenic differentiation was investigated using SEM imaging (Figure 10). The results indicate higher number of cells on both PCL and NW, however, cells on NW have extended morphologies with cellular extension that interact with the individual nanowires. In the higher magnification images of cells on NW, it is evident that the cells were attaching with individual nanowires that allows them to stretch long distances as they migrate across the surface. In addition, some of the ADSCs interact with the nanowires by embedding themselves between the grooves of grouped nanowires.

**Figure 11.**
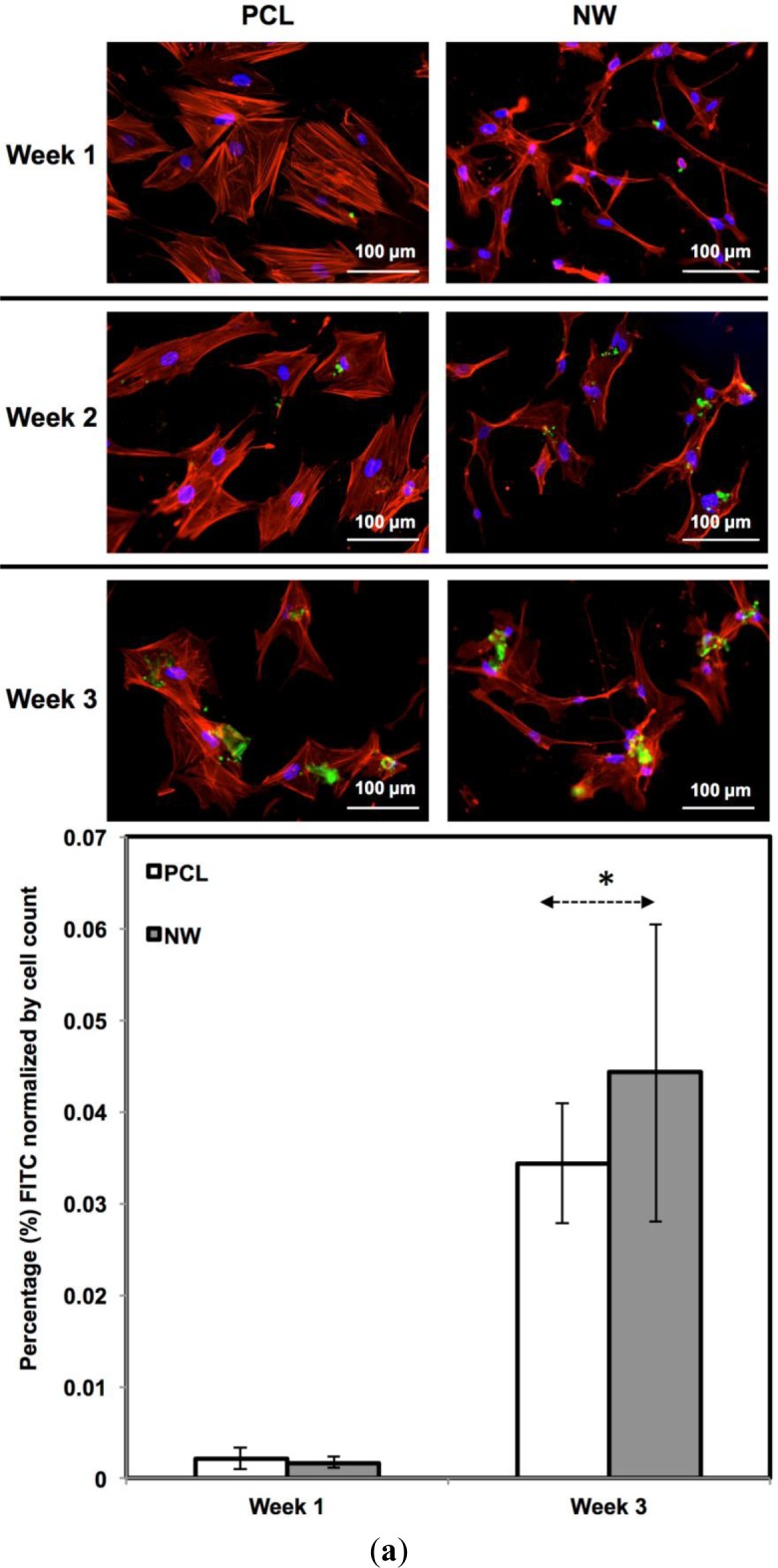

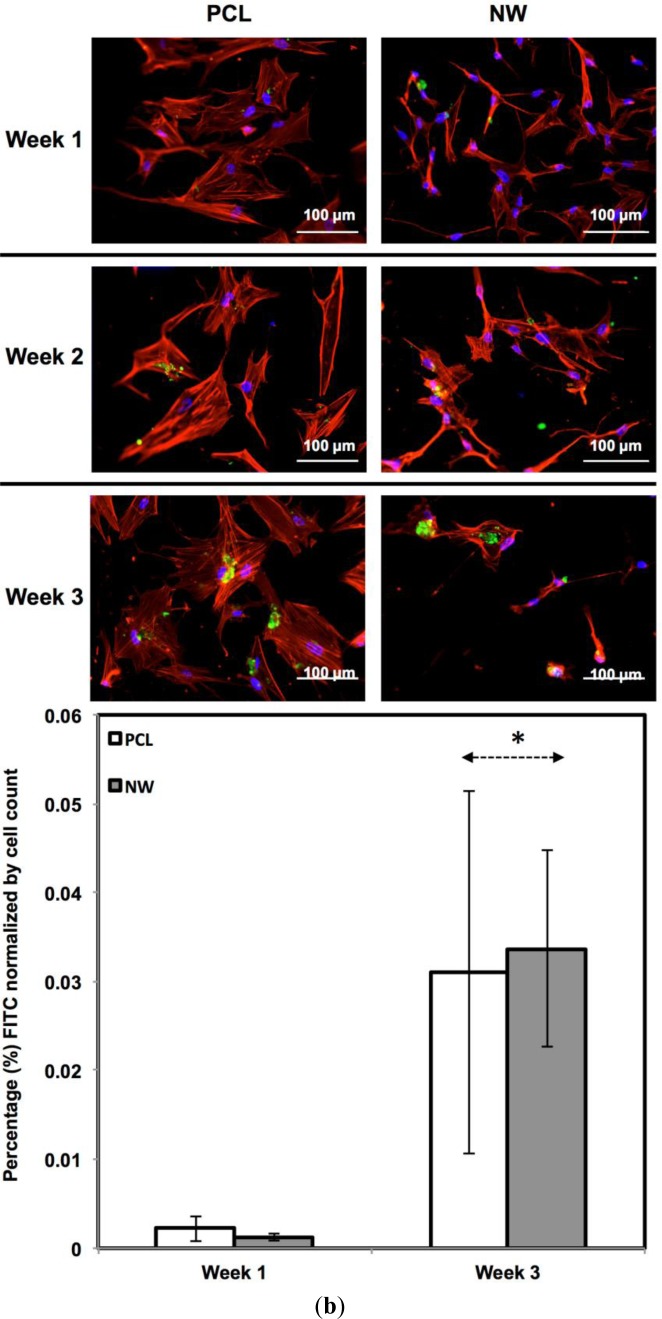
(**a**) Representative immunofluorescence images of ADSCs on PCL and NW for PPARγ (green), actin (red) and nuclei (blue) after 1, 2, and 3 weeks of culture in adipogenic conditions; (**b**) Representative fluorescence images of ADSCs on PCL and NW for adiponectin (green), actin (red) and nuclei (blue) after 1, 2, and 3 weeks of culture in adipogenic conditions. Percentage of FITC-labeled PPARγ (**a**) and adiponectin (**b**) were normalized by total number of cells within a particular image. Asterisks above Week 3 indicate statistical significance from Week 1 using p-value less than α = 0.05.

For surfaces cultured under adipogenic differentiation conditions, ADSCs on different surfaces were immuno-labeled for PPARγ and adiponectin (Figure 11a,b). PPARγ is a regulator of fatty acid storage and glucose metabolism. It also is known to stimulate lipid uptake and adipogenesis by fat cells. Adiponectin is a protein hormone secreted by mature adipocytes that regulates the metabolism of lipids. Adiponectin is also involved in regulating glucose metabolism and the breakdown of fatty acids. PPARγ and adiponectin were detected by FITC-labeled immunofluorescence during the 3 weeks ADSCs were cultured under adipogenic conditions.

The percentage of FITC detected during immunofluorescence was normalized by the number of cells on the particular image (Figure 11a,b). It was determined that for PPARγ expression, the protein was present on both surfaces during all three weeks of differentiation with very low levels on the first week. Both surfaces saw a significant increase in PPARγ from week 1 to week 3 with slightly more average expression on NW, however, not enough for it to be statistically different from PCL. Adiponectin had a very similar behavior to PPARγ. Adiponectin expression was detected on both surfaces during the first week even though the amount of FITC-labeled adiponectin is relatively low. After the third week of culture, both surfaces saw a significant increase in adiponectin with slightly more on NW even though PCL and NW are not statistically different.

**Figure 12.**
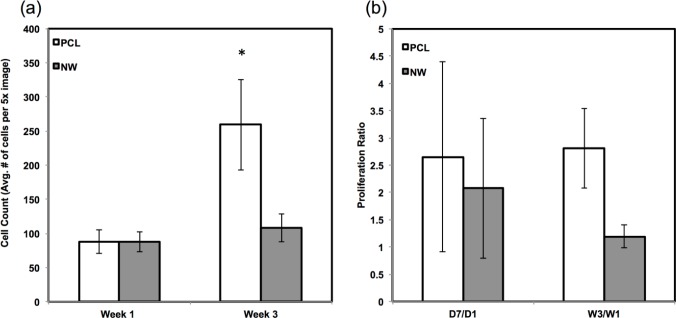
(**a**) Average cell counts on PCL and NW after 1 and 3 weeks of culture in adipogenic conditions. Cell nuclei were counted using DAPI fluorescence and ImageJ software [45]; (**b**) Comparisons of proliferation ratios between the growth period (D7/D1) and the differentiation period (W3/W1).

The amount of proliferation from week 1 to week 3 did not differ on NW, but increased significantly on PCL under adipogenic conditions (Figure 12a). The proliferation ratio for PCL during initial growth period (D7/D1) is similar to that during the differentiation period (W3/W1) (Figure 12b). However, the proliferation ratio of NW during growth period (D7/D1) is higher than that during the differentiation period (W3/W1) (Figure 12b). This indicated that the cells on NW are differentiating into adipogenic cells rather than proliferation unlike the cells on PCL.

**Figure 13.**
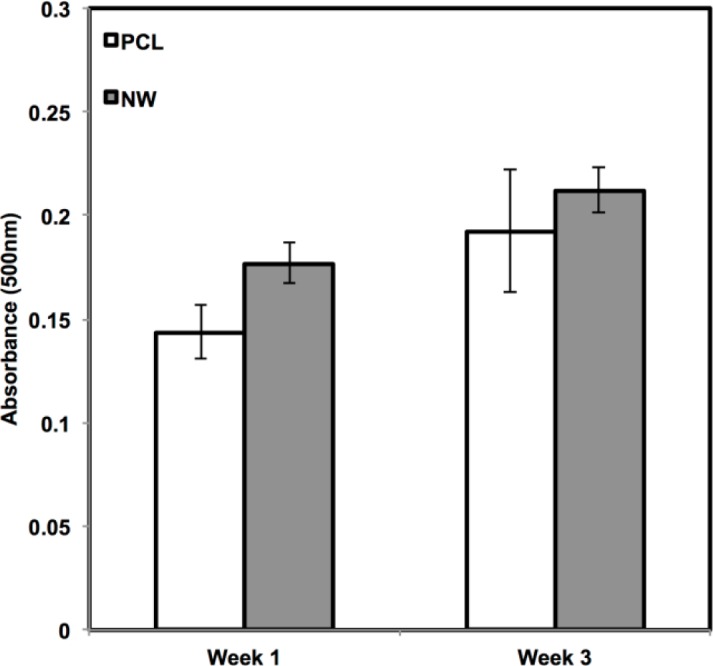
Oil red O (lipid) staining of PCL and NW after 1, 2 and 3 weeks of culture in adipogenic conditions as well as quantification of oil red O absorbance after 1 and 3 weeks of culture.

ADSCs on both surfaces were also stained using Oil Red O to detect for adipogenic differentiation and extracellular matrix production of lipids (Figure 13). Oil Red O is a fat soluble diazo dye that stains for triglycerides and lipids predominantly in frozen tissue sections and paraffin sections. However, it was more recently been used in cell culture and is becoming an increasingly common technique to detect for mature adipocytes during induced adipogenesis. After one week in adipogenic differentiation conditions, both surfaces stained for Oil Red O with more stain detected on NW in comparison with PCL. It is evident after three weeks of differentiation conditions that lipid production was increasing from the differentiating cells on both surfaces and trend continued with slightly more Oil Red O stain on NW. Oil Red O results seemed to follow immunofluorescence, which showed slightly more adipogenic expression on NW even though the number of cells and the rate of proliferation was lower than PCL.

**Figure 14.**
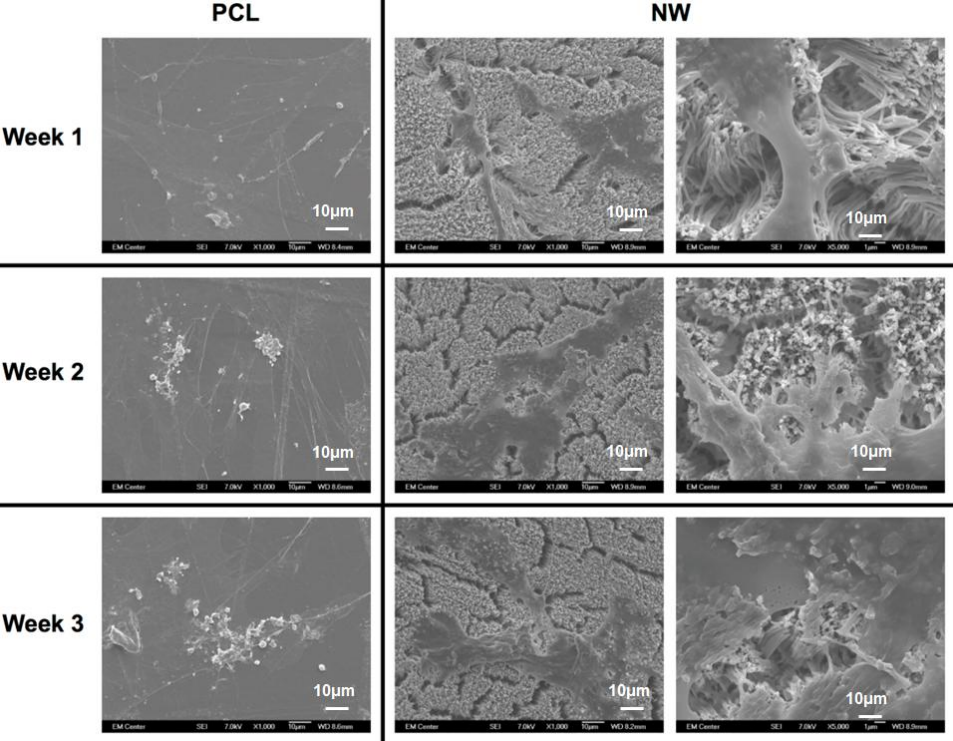
Representative SEM images of ADSCs on PCL and NW after 1, 2, and 3 weeks of culture in adipogenic conditions.

The cell morphology after 1 and 3 weeks of differentiation was investigated using SEM imaging (Figure 14). As with fluorescence imaging, the results from the images indicate higher number of cells on PCL with very little increase in the number of cells on NW. However, cells on NW have extended morphologies with cellular extension that interact with the individual nanowires.

## 3. Experimental Section

### 3.1. Fabrication of Smooth PCL and Nanowire Surfaces

A template synthesis technique was utilized for fabrication of polycaprolactone nanowire surfaces (notation: NW). Polycaprolactone powder with a molecular weight of 50,000 Da was sintered for 10 min at 115 °C. The sintered powder was allowed to cool to room temperature and smooth polycaprolactone discs (10 mm in diameter, notation: PCL) were punched using a standard aluminum punch. These discs were gravimetrically extruded though 100 nm diameter pores of inorganic aluminum oxide membranes (ANOPORE™) for 20 min just above the melting temperature at 70 °C to form NW. Differential scanning calorimetry (DSC, Fort Collins, CO, USA) was used to determine the exact melting temperature of the PCL prior to nanowire extrusion. The alumina membranes were dissolved in 1M NaOH for 75 min followed by profuse rinsing in DI water to release substrate bound nanowire surfaces. Scanning electron microscopy (SEM) (JEOL JSM-6500F SEM, Fort Collins, CO, USA) and energy dispersive X-ray spectroscopy (EDS, Fort Collins, CO, USA) were used to confirm the absence of alumina and NaOH on NW surfaces.

### 3.2. ADSC Culture on PCL and Nanowire Surfaces

Adult human ADSCs at passage 2 (Zen-Bio Inc., Durham, NC, USA) were expanded using standard cell culture techniques. All the cells used in this study were at passage 5 or below. Cells were detached using 0.25% Trypsin-EDTA and suspended in growth media consisting of DMEM with 10% fetal bovine serum (FBS, Atlanta Biologicals, Flowery Branch, GA, USA) and 1% penicillin/streptomycin (Sigma). All the surfaces were sterilized by incubating in 70% ethanol at room temperature followed by exposure to UV light for 30 min. Following sterilization, the surfaces were rinsed twice with warm phosphate buffered saline (PBS, Thermo Fisher Scientific Inc., Waltham, MA, USA). Cells were seeded on all surfaces in 48-well plates at a density of 5000 cells/well. The surfaces were incubated at 37 °C and 5% CO_2_ for the entire duration of the study. Half of the growth media was changed on day 4. On day 7, all the growth media was replaced with either chondrogenic differentiation media or adipogenic differentiation media or adipogenic differentiation media and the cells were cultured for up to 3 weeks.

Chondrogenic differentiation media consisted of growth media plus dexamethasone (10^−7^ M), ascorbic acid (50 mg/mL), ITS+ premix (1%) and TGF-β1 (10 ng/mL). Media was changed every other day for the entire duration of the culture.

Adipogenic differentiation media consisted of growth media plus dexamethasone (10^−7^ M), isobutylmethylxanthine (500 μM), biotin (33 μM), calcium pantothenate (17 μM) and human insulin (1 μM). Media was changed every other day for up to 7 days. After 7 days, the differentiation media was replaced with adipogenic maintenance media consisted of differentiation media without isobutylmethylxanthine and was changed every other day for the rest of duration of the culture.

### 3.3. ADSC Adhesion and Proliferation on PCL and Nanowire Surfaces

After 1, 4 and 7 days of initial culture, ADSC adhesion, proliferation, and spatial organization were investigated by staining the adhered cells with 5-Chloromethylfluorescein Diacetate (CMFDA–live cells), rhodamine-phalloidin (actin–cytoskeleton), and 4′,6-diamidino-2-phenylindole (DAPI–nucleus). At each time point, the surfaces were removed from the growth media, and incubated in the CMFDA stain at a concentration of 10 μM for 45 min in a 37 °C and 5% CO_2_ incubator. Next, the substrates were incubated for another 30 min at 37 °C and 5% CO_2_ in warm growth medium. The cells were then fixed with 3.7% formaldehyde for 15 min at room temperature. In order to permeabilize the cells, the surfaces were incubated in 1% Triton-X 100 for 3 min. The substrates were then incubated in rhodamine-phalloidin stain at a concentration of 5 μL/mL for 30 min. After 25 min of rhodamine-phalloidin staining, DAPI was added at a concentration of 300 mM. The substrates were then rinsed in PBS and imaged using a Zeiss Axioplan 2 fluorescence microscope. The number of adhered cells on all the surfaces was determined from 5× DAPI stained images by counting the individual nuclei using ImageJ software [45]. In addition to cell counts, cell shape factor was also determined using ImageJ software [45] with 10× rhodamine-phalloidin stained images. The cell shape factor was approximated by the ratio of cellular width to cellular length. The cellular width was defined as the diameter of the largest circle that would fit entirely within the cell and the cellular length was defined by the diameter of the smallest circle that encompassed the entire cell.

Cell viability was measured after 1, 4 and 7 days of initial culture (log phase growth) using a commercially available MTT assay kit (Sigma). Adhered cells were incubated at 37 °C for 3 h in a (3-[4,5-dimethylthiazol-2-yl]-2,5-diphenyl tetrazolium bromide (MTT) solution. Mitochondrial dehydrogenase of viable cells cleaves the tetrazolium ring leaving behind purple formazan crystals. The formazan crystals were dissolved in the MTT solvent. The optical density (OD) of the resulting solvent was measured at 570nm using a spectrophotometer (FLUOstar Omega; BMG Labtech, Durham, NC, USA). Background absorbance was measured at 690 nm and subtracted from the measured absorbance.

Material cytotoxicity was characterized after 1, 4 and 7 days of initial culture using a commercially available lactate dehydrogenase (LDH) cytotoxicity assay kit (Cayman Chemical). The protocol provided by the manufacturer was followed. Substrates were rapidly shaken on a horizontal shaker plate (1000 rpm) for 5 min at room temperature. The manufacturer-provided standards along with the substrate-exposed plasma samples were transferred to a 96 well plate. A reaction solution (96% V/V assay buffer, 1% V/V nicotinamide adenine dinucleotide (NAD+), 1% V/V Lactic Acid, 1% V/V iodonitrotetrazolium (INT), and 1% V/V LDH Diaphorase) was added in the amounts equal (1:1) to all standards and samples, and further incubated with gentle shaking on an orbital shaker for 30 min at room temperature. The absorbance of the solution was measured at a wavelength of 490 nm to determine the cytotoxic effects of the smooth PCL and nanowire substrates.

The morphology of the adhered ADSCs was investigated using SEM after 1, 4 and 7 days of culture. SEM was done to visualize how cells adhered and proliferated on the surfaces as well as how they interacted with the nanowire morphology. In brief, the cells were fixed in a solution of 3% glutaraldehyde, 0.1 M sodium cacodylate, and 0.1 M sucrose for 45 min. The surfaces were then incubated in a buffer containing 0.1 M sodium cacodylate and 0.1 M sucrose. After fixation, the cells were dehydrated by incubating the surfaces in increasing concentrations of ethanol (35%, 50%, 70%, 100%) for 10 min each. The surfaces were further dehydrated by incubating them in hexamethyldisilazane for 10 min. The surfaces were stored in a desiccator until examination using SEM. The surfaces were sputter coated with 10 nm of gold and imaged using at a voltage of 7 kV.

### 3.4. ADSC Chondrogenic Differentiation

ADSC responses to the different surfaces were evaluated after providing the cells with chonodrogenic differentiation media. After 1, 2 and 3 weeks of culture, ADSCs on different surfaces were immuno-labeled for Sox9 and Collagen 2 (Col2). Cells were fixed and permeabilized as described earlier. All the surfaces were incubated with 10% bovine serum albumin for 30 min at room temperature to prevent nonspecific binding. After rinsing in PBS, the surfaces were incubated with either anti-Sox9 primary antibody (1:100 in PBS, purified goat polyclonal antibody of human origin, Santa Cruz Biotechnology) or anti-Col2 primary antibody (1:100 in PBS, purified goat polyclonal antibody of mouse origin, Santa Cruz Biotechnology) for 1 hr at room temperature. Following primary antibody incubation, surfaces were rinsed three times with PBS at an interval of 5 min each. The surfaces were then incubated in FITC-labeled secondary antibodies for Sox9 and Col2 (1:200 donkey antigoat IgG, Santa Cruz Biotechnology) for 45 min in the dark. Finally, the surfaces were rinsed twice in PBS and incubated in rhodamine-phalloidin stain at a concentration of 5 μL/mL for 30 min. After 25 min of rhodamine-phalloidin staining, DAPI was added at a concentration of 300 nM. The surfaces were then rinsed in PBS and visualized using a Zeiss Axioplan 2 fluorescence microscope. The number of adhered cells on all the surfaces after 1 and 3 weeks of culture was determined from 5× DAPI stained images by counting the individual nuclei using ImageJ software [45]. Using the cell counts, cell proliferation ratio from day y to day 1 and week 7 to week 1 was calculated.

Glycosaminoglycan formation on the surfaces was detected using alcian blue staining after 1, 2, and 3 weeks of culture. All surfaces were rinsed twice in PBS followed by fixation in cold (4 °C) acetone:methanol solution for 3 min. Substrates were transferred to a 1% alcian blue solution in 3% acetic acid. The surfaces were incubated at room temperature in alcian blue for 30 min followed by three rinses in 3% acetic acid for 2 min each. After rinsing in deionized water for 2 min, the surfaces were allowed to dry for imaging or placed in a 1% sodium dodecyl sulfate solution for 30 min on a 200 rpm shaker plate to solubilize the alcian blue stain. Absorbance of the solubilized solution was measured at 605 nm.

The morphology of adhered ADSCs on surfaces was investigated using SEM after 1, 2 and 3 weeks of culture using the method described in previous section.

### 3.5. ADSC Adipogenic Differentiation

ADSC responses to the different surfaces were evaluated after providing the cells with adipogenic differentiation media followed by adipogenic maintenance media. After 1, 2 and 3 weeks of culture, ADSCs on different surfaces were immuno-labeled for PPARγ and adiponectin (Acrp30). Cells were fixed and permeabilized as described earlier. All the surfaces were incubated with 10% bovine serum albumin for 30 min at room temperature to prevent nonspecific binding. After rinsing in PBS, the surfaces were incubated with either anti-PPARY primary antibody (1:100 in PBS, purified goat polyclonal antibody of human origin, Santa Cruz Biotechnology) or anti-Acrp30 primary antibody (1:100 in PBS, purified goat polyclonal antibody of mouse origin, Santa Cruz Biotechnology) for 1 h at room temperature. Following primary antibody incubation, surfaces were rinsed three times with PBS at an interval of 5 min each. The surfaces were then incubated in FITC-labeled secondary antibodies for PPARγ and Acrp30 (1:200 donkey antigoatIgG, Santa Cruz Biotechnology) for 45 min in the dark. Finally, the surfaces were rinsed twice in PBS and incubated in rhodamine-phalloidin stain at a concentration of 5 μL/mL for 30min. After 25 min of rhodamine-phalloidin staining, DAPI was added at a concentration of 300 nM. The surfaces were then rinsed in PBS and visualized using a Zeiss Axioplan 2 fluorescence microscope. The number of adhered cells on all the surfaces after 1 and 3 weeks of culture was determined from 5× DAPI stained images by counting the individual nuclei using ImageJ software [45]. Using the cell counts, cell proliferation ratio from day y to day 1 and week 7 to week 1 was calculated.

Lipid formation on the surfaces was detected using Oil Red O staining after 1, 2, and 3 weeks of culture. All surfaces were rinsed twice in PBS followed by fixation in 10% formalin for 1 h. Surfaces were rinsed in 60% isopropanol and transferred to a 0.5% Oil Red O solution in 60% isopropanol. The surfaces were incubated at room temperature in Oil Red O for 15 min followed by three rinses in deionized water for 2 min each. After rinsing, the surfaces were placed in a 100% ispropanol for 30 min on a 200 rpm shaker plate to solubilize the Oil Red O stain. Absorbance of the solubilized solution was measured at 500 nm.

The morphology of adhered ADSCs on surfaces was investigated using SEM after 1, 2 and 3 weeks of culture using the method described in previous section.

### 3.6. Statistical Analysis

Data within the graphs are expressed as the average count and the standard deviation of the mean. All the quantitative results were analyzed using paired *t*-test or one-way analysis of variance (ANOVA). Multiple comparisons are tested using Tukey’s HSD. Statistical significance was considered at *p* < 0.05. All qualitative methods used *n*_min_ = 3 and all quantitative methods used *n*_min_ = 5. Experiments were repeated at least three times with three different cell culture populations.

## 4. Conclusions

This study investigated the differentiation potential of ADSCs on polycaprolactone nanowire into chondrogenic and adipogenic phenotypes. ADSC adhesion, proliferation, viability, and morphology were investigated for up to 7 days of culture using fluorescence microscopy imaging, a cell viability assay, and SEM. The results show that the cells on both surfaces, PCL and NW, promote adhesion and proliferation, however the cells on NW have much more elongated morphologies. Differentiation was investigated after the 7 day growth period for up to 3 weeks of culture using immunofluorescence imaging, histological staining, and SEM. On NW, more cells appear to have differentiated into adipogenic phenotypes, whereas fewer cell appear to have differentiated into chondrogenic phenotypes as compared to cells on PCL. Expression of adipogenic marker proteins, PPARγ and adiponectin, increased, whereas there was no change in expression of chongrogenic proteins, sox9 and col2, over the 3 weeks of culture on the NW. In addition, chondrogenic histological staining using alcian blue, indicated lower GAG presence on NW as compared to PCL. Therefore, the results indicate that the nanowire architecture may provide a more favorable template for adipogenic differentiation and less favorable for chondrogenic differentiation of ADSCs. Further studies are now being conducted to determine the differentiation potential of ADSCs on nanowire surfaces without the aid of differentiation supplements to understand how the cells will respond to the nanoarchitechture alone over a long period of time.
